# Complete genome sequences of five *Ackermannviridae* that infect *Enterobacteriaceae* hosts

**DOI:** 10.1128/mra.00950-23

**Published:** 2024-02-07

**Authors:** Evan B. Harris, Laura B. Anthony, Sakhawat Ali, Hannah Atkin, Lucy C. Bowden, Steven W. Brugger, Emille L. Carr, Nathaniel Eberhard, Samuel Flor, Rochelle K. Gaertner, Austen Gleave, David Hess, Trevor Hoggan, Elisa Correa Lazaro, Katherine Leonard, Trek Lewis, Colleen R. Newey, Joshua Ramsey, Kayla R. Sajous, Daniel Schaeffer, Tyson Stoker, Sierra Stump, Daniel W. Thompson, Rachel Weyland, Julianne H. Grose

**Affiliations:** 1 Department of Microbiology and Molecular Biology, Brigham Young University, Provo, Utah, USA; Portland State University, Portland, Oregon, USA

**Keywords:** *Enterbacteriaceae*, bacteriophages, *ViO1*, Sajous1, FrontPhageNews, ChubbyThor, AR2819, SilasIsHot, *Salmonella*, *Shigella*

## Abstract

This announcement contains the whole genome sequences of five *Ackermannviridae* that infect members of the *Enterobacteriaceae* family of bacteria. Four of the five phages were isolated using *Salmonella enterica* serovar Typhimurium as a bacterial host: AR2819, Sajous1, SilasIsHot, and FrontPhageNews. ChubbyThor was isolated using *Shigella boydii*.

## ANNOUNCEMENT

The *Enterobacteriaceae* bacterial family is composed of Gram-negative facultative anaerobes and includes common pathogens such as *Salmonella enterica* serovar Typhimurium*, Klebsiella aerogenes, Escherichia coli,* and many others. Bacteriophages (phages) are an essential driver of bacterial evolution, including pathogenic traits, due to their prevalence, propensity to transfer genetic elements between strains, ability to persist as prophages within bacterial genomes, and lytic activity (1). This announcement contains the whole genome sequences of five related bacteriophages that infect members of the *Enterobacteriaceae*.

All five phages (Table 1) were isolated from effluent sewage collected from water treatment plants in the western United States. Four of the five phages were isolated using *Salmonella enterica* serovar Typhimurium LT2 as a bacterial host: AR2819, Sajous1, SilasIsHot, and FrontPhageNews. The final phage, ChubbyThor, was isolated using *Shigella boydi* Ewing (ATCC 9207). Briefly, effluent sewage was incubated with bacterial culture and LB nutrient broth at 37°C for 2 days prior to pelleting bacterial debris (8,000 rpm for 20 min) and plating in LB top agar. Single plaques were then picked and replated alongside bacteria in top agar. This plaque purification was repeated three times wherein all five phages appear to be lytic due to high concentration of clear plaques. DNA was purified from lysates containing at least 10^8^ PFU/mL using the Phage DNA Isolation Kit (Norgen Biotek, Canada) and prepared for Illumina iSeq 150 bp paired-end sequencing using the NEBNext Ultra II FS kit (New England Biolabs). Resulting reads were assembled *de novo* with automatic trimming using Geneious version R11 (2) at default settings. The phage genomes circularized upon assembly, and each genome was annotated using both DNAmaster (3) and GeneMark (4) and was deposited in NCBI GenBank (see Table 1). NCBI Genbank nucleotide database BLASTN analysis (5) as of 6 October 2023 revealed that all five phages display similarity in genome size and nucleotide identity to previously characterized *Enterobacteriaceae* phage cluster *ViO1* (6), which has been classified in the *Ackermannviridae* family by the International Committee on Taxonomy of Viruses (7). Phages Sajous1 and AR2819 are most like *Salmonella* phage Guerrero (accession no. OP610151) by BLASTN, sharing a 98.6% and 99.7% nucleotide identity, respectively, over 98% of the genome. FrontPhageNews is most like Sajous1 by BLASTN, with 86.66% identity over 95% of the genome. SilasIsHot is most like *Salmonella* phage vB_SenM_UTK0004 (OQ359883) with a 98.35% identity over 94% of the genome, while ChubbyThor is most like *Shigella* phage MK-13 (NC_049455.1) with a percent identity of 96.56% over 88% of the genome. The increased GC content of ChubbyThor, which sits at 50.1% as compared to the other phages that range between 44.61% and 45.13% GC content, may reflect host preference. Further characterization of these phages is currently underway and may help increase our understanding of *Enterobacteriaceae* evolution.

**TABLE 1:** Sequencing summary and properties of five *Ackermannviridae* that infect *Enterobacteriaceae*

Phage name	Original bacterial host	Sewage sampling location coordinates	Sample acquisition date	GenBank accession no.	SRA accession no.	Total no. of reads	Fold coverage range (mean)	Length (bp)	GC %^*a*^
AR2819	*Salmonella* Typhimurium	40.2729°N, 111.7382°W	10 September 2019	MW021753	SRR22024852	437,180	89–699 (284.6)	156,899	44.97
SilasIsHot	*Salmonella* Typhimurium	40.9012°N, 111.9302°W	10 September 2019	MW021760	SRR22024849	114,525	1–373 (235.9)	160,559	45.13
Sajous1	*Salmonella* Typhimurium	42.2134°N, 111.6500°W	10 September 2019	MW021757	SRR22024851	37,3801	35–250 (214.6)	157,255	44.86
FrontPhageNews	*Salmonella* Typhimurium	40.7418°N, 73.9883°W	13 May 2020	MW021754	SRR22024850	60,142	24–97 (55.7)	157,832	44.61
ChubbyThor	*Shigella boydii*	33.4152°N, 111.8315°W	1 September 2018	OL615013	SRR11575116	827,950	931 (737.9)	159,319	50.1

^*a*^ GC content was calculated using the GC-profile tool (8) with a halting parameter of 50 and 1,000 minimum segment length per recommendations provided with the tool.

## Data Availability

This whole genome sequencing project has been deposited in GenBank under two accession numbers, accession no. PRJNA891723 for phages AR2819 (MW021753), SilasisHot (MW021760), Sajous1 (MW021757), and FrontPhageNews (MW021754) and accession no. PRJNA626377 for phage ChubbyThor (OL615013). The versions described in this paper are the first versions, PRJNA891723 and PRJNA626377. See Table 1 for SRA accession numbers.

## References

[1] Klimenko AI, Matushkin YG, Kolchanov NA, Lashin SA. 2016. Bacteriophages affect evolution of bacterial communities in spacially distributed habitats: a simulation study. BMC Microbiol 16:67. doi:10.1186/s12866-016-0677-8 27079662 PMC4831195

[2] Kearse M, Moir R, Wilson A, Stones-Havas S, Cheung M, Sturrock S, Buxton S, Cooper A, Markowitz S, Duran C, Thierer T, Ashton B, Meintjes P, Drummond A. 2012. Geneious basic: an integrated and extendable desktop software platform for the organization and analysis of sequence data. Bioinformatics 28:1647–1649. doi:10.1093/bioinformatics/bts199 22543367 PMC3371832

[3] Lawrence J. 2007. DNA Master. Available from: http://cobamide2.bio.pitt.edu/

[4] Besemer J, Lomsadze A, Borodovsky M. 2001. Genemarks: a self-training method for prediction of gene starts in microbial genomes. implications for finding sequence motifs in regulatory regions. Nucleic Acids Res 29:2607–2618. doi:10.1093/nar/29.12.2607 11410670 PMC55746

[5] Altschul SF, Gish W, Miller W, Myers EW, Lipman DJ. 1990. Basic local alignment search tool. J Mol Biol 215:403–410. doi:10.1016/S0022-2836(05)80360-2 2231712

[6] Grose JH, Casjens SR. 2014. Understanding the enormous diversity of bacteriophages: the tailed phages that infect the bacterial family Enterobacteriaceae. Virology 468–470:421–443. doi:10.1016/j.virol.2014.08.024 PMC4301999 25240328

[7] Lefkowitz EJ, Dempsey DM, Hendrickson RC, Orton RJ, Siddell SG, Smith DB. 2018. Virus Taxonomy: The database of the International Committee on 84 Taxonomy of viruses (ICTV. Nucleic Acids Res 46:D708–D717. doi:10.1093/nar/gkx932 29040670 PMC5753373

[8] Gao F, Zhang C-T. 2006. GC-profile: a web-based tool for visualizing and analyzing the variation of GC content in genomic sequences. Nucleic Acids Res 34:W686–91. doi:10.1093/nar/gkl040 16845098 PMC1538862

